# Reduced RAN Expression and Disrupted Transport between Cytoplasm and Nucleus; A Key Event in Alzheimer’s Disease Pathophysiology

**DOI:** 10.1371/journal.pone.0053349

**Published:** 2013-01-08

**Authors:** Diego Mastroeni, Leonidas Chouliaras, Andrew Grover, Winnie S. Liang, Kevin Hauns, Joseph Rogers, Paul D. Coleman

**Affiliations:** 1 L. J. Roberts Alzheimer’s Disease Center, Banner Sun Health Research Institute, Sun City, Arizona, United States of America; 2 School for Mental Health and Neuroscience (MHeNS), Department of Psychiatry and Neuropsychology, Faculty of Health, Medicine and Life Sciences, European Graduate School of Neuroscience (EURON), Maastricht University Medical Centre, Maastricht, The Netherlands; 3 Collaborative Sequencing Center, Translational Genomics Research Institute, Phoenix, Arizona, United States of America; Univ. Kentucky, United States of America

## Abstract

Transcription of DNA is essential for cell maintenance and survival; inappropriate localization of proteins that are involved in transcription would be catastrophic. In Alzheimer’s disease brains, and *in vitro* studies, we have found qualitative and quantitative deficits in transport into the nucleus of DNA methyltransferase 1 (DNMT1) and RNA polymerase II (RNA pol II), accompanied by their abnormal sequestration in the cytoplasm. RAN (*RA*s-related *N*uclear protein) knockdown, by siRNA and oligomeric Aβ42 treatment in neurons, replicate human data which indicate that transport disruption in AD may be mechanistically linked to reduced expression of RAN, a pivotal molecule in nucleocytoplasmic transport. *In vitro* studies also indicate a significant role for oligomeric Aβ42 in the observed phenomena. We propose a model in which reduced transcription regulators in the nucleus and their increased presence in the cytoplasm may lead to many of the cellular manifestations of Alzheimer’s disease.

## Introduction

Normal cell function requires constant exchange of molecules between the nucleus and the cytoplasm. Classically, RNA from the nucleus is packaged and transported to the cytoplasm for translation, and the resulting, newly-synthesized molecules either remain in the cytoplasmic compartment or are transported back into the nucleus to participate in a variety of functions. Virtually all nuclear proteins begin their existence in the cytoplasm, and their failure to be translocated back to the nucleus could be as catastrophic as their failure to be synthesized. Nucleocytoplasmic transport in both directions is mediated by transport proteins and macromolecules that carry molecules through the nuclear pore complex, a process that depends on the active participation of the pivotal molecule RAN [1].

Epigenetic and other highly-conserved transcription-related factors are among important proteins that depend on RAN-mediated transport for appropriate nuclear localization [2], [3]. DNA methylation by DNA methyltransferases (DNMT1 for example), can alter binding of transcription factors to their target genes, as well as recruit methyl-CpG-binding domain (MBD) proteins to the locus [4]. This, in turn, may recruit other chromatin remodeling proteins such as histone deacetylase 1 (HDAC1), thereby modifying histone proteins and transcriptional access [5]. Likewise, RNA pol II, which is essential for transcribing RNA also requires RAN-mediated transport directly, or indirectly, in order to fulfill its functional roles [6], [7].

Several previous studies have suggested deficits in Alzheimer disease (AD) of molecules related to transport between the nucleus and the cytoplasm [8]–[10]. These reports led us to hypothesize disrupted transport of transcription-related molecules between the cytoplasm and the nucleus in AD. In view of the emerging evidence of the importance of epigenetic molecules in regulating gene expression in AD[11]–[13], our tests of this hypothesis concentrated on a fundamental epigenetic molecule, DNMT1, as well as one other molecule representative of an additional aspect of regulation of transcription, RNA pol II.

Examination of human and *in vitro* samples using immunohistochemistry, Western blots, *in situ* hybridization, gene expression microarrays and siRNA revealed a potentially catastrophic failure of transport into the nucleus of DNMT1 and RNA pol II in pathologically-vulnerable AD neurons. We show, for the first time, decreased appearance of these molecules in the nucleus and their cytoplasmic sequestering. Our data also indicate that this phenomenon may be causally linked to reduced protein and mRNA expression of RAN, a pivotal component in the transport of molecules between the cytoplasm and nucleus, and that oligomeric Aβ42 plays a significant role in the phenomena we describe.

## Materials and Methods

### Ethics Statement

Written informed consent for autopsy was obtained for all cases in compliance with institutional guidelines of Banner Sun Health Research Institute. Banner Sun Health Research Institute review board approved this study including recruitment, enrollment, and autopsy procedures. All donors and their respective next-of-kin consented to brain autopsy for the purpose of research analysis as participants in the Banner Sun Health Research Institute autopsy program. The human brain tissue used in this manuscript was from routine existing autopsies, which fully qualifies for 4C exemption by NIH guidelines.

### 1.1. Subjects, Brain Samples and Cells

Samples of human limbic cortex, and cerebellum were secured from AD and ND brains obtained at autopsy at the Banner Sun Health Research Institute Tissue Bank. Cognitive status of all cases was evaluated antemortem by board-certified neurologists, and postmortem examination by a board-certified neuropathologist resulting in a consensus diagnosis using standard NIH Alzheimer’s Center criteria for AD or neurologically normal, non-demented elderly control (ND). The AD and ND groups were well matched for age (AD: 83+/−3.3 years; ND: 84±2.4 years), gender (3 females and 2 males in each group), and postmortem interval (PMI) (AD: 3 hours 12 min +/−9 min; ND: 3 hours 48 minutes +/−8 min). For ***in vitro*** **studies**, SK-N-BE(2) neuroblastoma cells (ATCC) were reconstituted and maintained following ATCC guidelines. Cells were maintained in a humidified 37°C incubator with 5% CO_2_, and were supplied with complete DMEM (500 ml DMEM with high glucose, minus phenol red (Invitrogen-Gibco)50 ml FBS (Gemini Bio-Products; West Sacramento, CA), 10 ml HEPES (Irvine Scientific, Santa Ana, CA.), 5 ml sodium pyruvate (Mediatech Cellgro), 5 ml penicillin/streptomycin (Invitrogen-Gibco), and 0.5 ml gentamycin (Irvine Scientific) every three days until experiments were performed (approx. 1 week after initial platting). The general health of the neurons before and after treatment was tested using both calcein AM (Invitrogen) and LDH assays (Invitrogen) (Fig S1), which showed excellent health.

#### Affymetrix array studies

RNA was isolated from laser captured pyramidal single neurons from postmortem human samples of CA1 of the hippocampus, superior frontal gyrus, and visual cortex. Gene expression was analyzed using Affymetrix Human Genome U133 plus 2.0 microarrays. The AD (n = 10) and ND (n = 10) groups were well matched for age (AD: 77.8+/−4 years; ND: 81.5+/−1.2 years), gender (AD: 3 females and 7 males, ND: 4 females and 6 males). See section 1.4. for further details.

### 1.2. Immunohistochemistry/Immunocytochemistry

Brain tissue or cells were fixed in PFA, washed in phosphate buffer (PB). Tissue samples were sectioned at 40 µm on a cryostat, and stored in a cryoprotectant solution of 33% glycol/33% glycerol/33% PB at −20°C until required for experiments. Sections were washed, incubated in 10 mM citrate buffer (antigen retrieval) for 10 min at 95°C, washed, blocked in 1% hydrogen peroxide followed by 1 h incubation in 3% bovine serum albumin (BSA), and incubated at 4°C overnight in primary antibody solutions containing 0.25% BSA. Sources and dilutions for antibodies are given in Table 1. After incubation, the sections were washed, incubated in biotinylated, species-specific secondary antibodies (Vector), and incubated in avidin-biotin complex (Pierce). Following incubation with secondary antibodies, the sections were washed and immersed in DAB solution for no longer than 10 min, followed by two quick rinses in 50 mM Tris to stop the reaction. AD and ND sections were immunoreacted simultaneously using netwells in well-less plates. Sections were mounted with Permount (Pierce). For fluorescence microscopy, the sections were washed 3X, blocked with either 3% normal goat serum or 3% BSA, and incubated for 1 h. After further washing, sections were incubated in primary antibody, washed again, and incubated in species–specific, fluorophore-conjugated secondary antibodies (Molecular Probes). After a final wash, the sections were mounted, taken through Sudan Black to reduce autofluorescence, and coverslipped with Vectashield (Vector). Deletion of primary antibody or incubation with pre-immune serum resulted in abolition of specific immunoreactivity in all cases (data not shown). Adjacent serial sections were stained with cresyl violet, or within sections with neutral red for structural visualization. For some sections, nuclei were counterstained with 4′,6′-diamidino-2-phenylindole (DAPI) (Invitrogen), a nuclear counterstain, before mounting.

**Table 1:** The antibodies used, sources, dilutions and recognition motif.

Antibody	Host	Dilution	Source/Catalogue#	Recognition Sequence
Anti- RNA pol II	Rabbit polyclonal	1∶1000	Millipore/ABE30	RNA polymerase II subunit B1
Anti-RAN	Rabbit polyclonal	1∶1000	Abcam/53775	Synthetic peptide to human RAN (full length)
Anti-Oligomer	Rabbit polyclonal	1∶10,000	Invitrogen/AHB0052	Recognizes Oligomers
Anti-DNMT1	Rabbit polyclonal	1∶500	Abcam/ab19905	Within residues 100–200

Immunostained tissue sections were examined on Olympus IX51 and Olympus IX70 microscopes equipped with epifluorescence illumination or with confocal laser scanning using argon and krypton lasers (Olympus IX70). The findings were documented photographically with an Olympus DP-71 color digital camera or, for confocal microscopy, by Fluoview software (Olympus). Following photographical imaging, cellular localization was quantified using Image J software.

### 1.3. Western Blot Analysis

For Western blots, tissue was cut into 1 cm cubed pieces and white matter was carefully removed under a dissecting microscope, reserving the grey matter only for processing. After dissection, tissue was lysed in a solution containing 20 mM Tris pH7.5, 0.5% Nonident (Sigma), 1 mM EDTA (Sigma), 0.1 M NaCl (Sigma), 1 mM PMSF (Sigma), Sigma protease inhibitors 1, 2, and complete protease inhibitor cocktail (Roche). Protein concentrations were determined by BCA assay (Pierce) using bovine serum albumin as the standard. A total of 20 ug of sample protein was combined with Laemmli sample buffer for separation by SDS-PAGE, followed by transfer to PVDF membrane (Bio-Rad). Membranes were blocked using 5% non-fat dry milk and probed with primary antibodies (Table 1). After incubation with primary antibody, membranes were washed, incubated with secondary antibody, washed again, reacted with chemiluminescence substrate (Pierce), imaged on an Alpha Ease detection system, and analyzed using AlphaEaseFC software (Alpha Innotech).

### 1.4. RAN and RAN Binding Proteins in Gene Expression Microarray

To extend the RAN data to selected RAN related transcripts, we mined a recent, collaborative, gene expression microarray study in which AD and ND cortical and hippocampal neurons were evaluated after laser capture microdissection. Detailed methods for the study, which employed autopsy specimens from our Institution’s brain bank and the brain bank at Washington University, have been previously published [14]. Briefly, RNA was isolated from approximately 500 laser-captured pyramidal neurons from the superior frontal gyrus (BA 10 and 11), visual cortex (BA 17), and CA1 of the hippocampus. Following amplification 10 µg of fragmented cRNA was hybridized to an Affymetrix Human Genome U133 plus 2.0 Array. All genes that did not meet a 10% present call threshold were removed by Genespring GX 7.3 Expression Analysis software (Agilent, technologies: Palo Alto, CA).

### 1.5. RAN mRNA Probe Construction and *In situ* Hybridization

#### Probe construction

RAN DNA oligonucleotide template was designed using Primer 3 software (http://frodo.wi.mit.edu/primer3/). Palindromic sequences were omitted using palindromic sequence finder (http://biophp.org/minitools/find_palindromes/demo.php), and sequence combinations stretching across exon-exon boundaries were omitted. The resulting templates were 5′-TGGTTGGTGATGGTGGTACTGGAAAAACGACCTTCGTGAAACGTCA TTTGACTGGTGAATTTGAGAAGAAGTATGTAGCCACCTTGGGTGTTGAGGTTCAT-3′ and 5′-AAGAAGAATC TTCAGTACTACGACATT-3′ (Sigma). Both sequences required the addition of an eight nucleotide sequence to the 3′ end of the oligonucleotide (5′-CCTGTCTC-3″). Sense probes were constructed as negative controls. The mRNA probes for sense and anti-sense were constructed following the manufacturer’s instructions, using the mirVana miRNA Probe Construction Kit (Applied Biosystems).

#### *In situ* hybridization

20 µm hippocampal AD (n = 3) and ND (n = 3) brain sections were mounted onto plus slides and allowed to dry overnight, immersed in a 0.007% pre-warmed 600 mAU/ml solution of proteinase K (Qiagen), and washed in DEPC ddh20 followed by the addition of 125 µl acetic anhydride in 0.1 M TEA. After incubating for 15 min at RT, sections were washed in 2×SSC (saline sodium citrate), incubated in pre-hybridization solution, rinsed, and reacted with RAN-specific biotinylated mRNA probe in hybridization solution. Tissue was then incubated for 16 h at 42°C in a humid chamber. After incubation, slides were washed, incubated with 50% formamide, rinsed in 2×SSC, incubated in 100 µg/ml Rnase A solution, washed, and incubated in a series of SSC buffer. For staining, samples were treated as stated in section 1.2., with the exception that Streptavidin-conjugated secondary antibodies were employed to detect the biotinylated probes.

### 1.6. siRNA RAN Knockdown

SK-N-BE(2) neuroblastoma cells were seeded at a density of 15,000 cells/well in 12-well plates using antibiotic-free medium. The next day, cell culture medium was removed, replaced with Opti-MEM (Invitrogen), and cultures were placed in a 37°C incubator with 5% CO_2_. ON-TARGETplus Non-targeting Pool negative control siRNA (Dharmacon) and ON-TARGETplus SMARTpool siRNA to human RAN (Dharmacon) and cells were resuspended in nuclease free H_2_O and diluted in Opti-MEM to various concentrations. The manufacture’s protocol for Lipofectamine 2000 (Invitrogen) RNAi transfection mix was employed throughout, except for the Lipofectamine 2000 volume, which was increased to 2 µL. Opti-MEM was then removed from the SK-N-BE(2) cells and each siRNA transfection complex was added to a specific well of cells. The cells were rocked every 10 min for 0.5 h in a 37°C incubator with 5% CO_2_. After 0.5 h the cells were overlaid with 900 µL Opti-MEM for a final volume of 1 mL. Final concentrations of negative control siRNA and RAN siRNA are indicated in the Figure legend. After 6 h the Opti-MEM medium was removed and replaced with DMEM (Gibco) supplemented with 10% fetal bovine serum. Post transfection of RAN and control siRNA took place for 48 h in a 37°C incubator with 5% CO_2_. Following experimental manipulation, cells were immunoreacted against antibodies (Table 1), following the methods stated in section 1.2. Cells were examined using epi-fluorescences (IX-71) microscopy. Experiments were performed in triplicate.

### 1.7. *In vitro* Amyloid Beta 42 Treatment

SK-N-BE(2) neuroblastoma cells were treated as described in section 1.1. prior to the addition of higher oligomeric or lower oligomeric amyloid beta 42 (Bachem). Amyloid beta 42 peptides were reconstituted in 0.5% ammonium hydroxide, and were immediately frozen down (lower molecular weight oligomeric Aβ42), or allowed to sit at room temperature for 96 hours (higher molecular weight oligomeric Aβ42). Western blot analysis using oligomeric antibody A11 (Sigma), revealed the presence of oligomers in both preparations. Amyloid beta 42 peptides were diluted in serum free Opti-Mem(Gibco) at an experimentally determined concentration of 1 uM. Cells were incubated for 36 hours in triplicate and immunoreacted against antibodies (Table 1), following the methods stated in section 1.2. Cells were examined using confocal and epi-fluorescences microscopy and quantified using image J software.

### 1.8. Statistical Analysis

#### Tissue and cells

**Figure 1:**
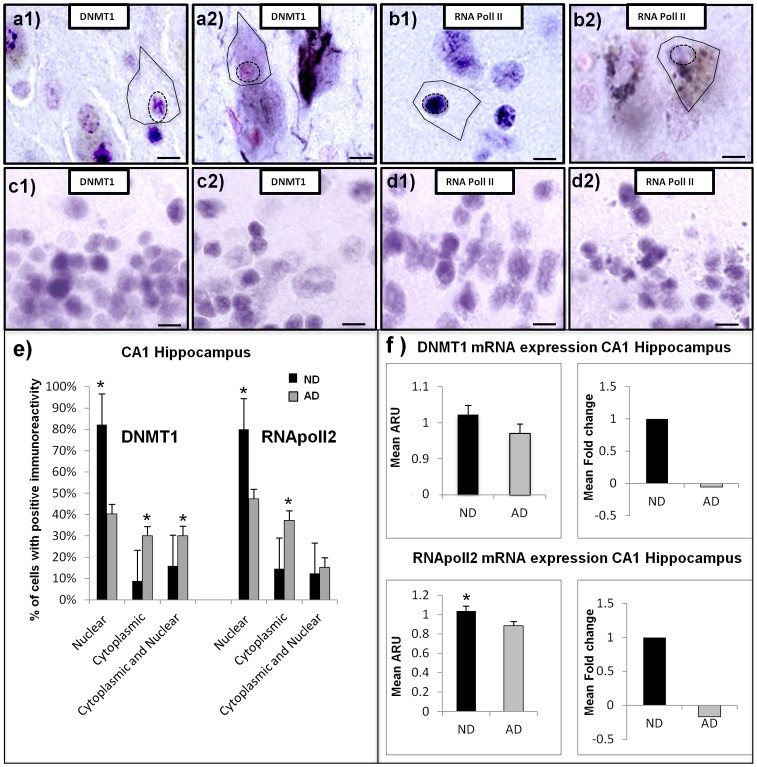
Cellular localization of epigenetic and other transcription-related molecules in CA1 of the hippocampus from AD and ND cases. All tissue samples were counterstained with neutral red for cell layer and cell landmark verification. (a1) high power micrograph of CA1 labeled with an antibody to DNMT1 in a typical ND case, showing appropriate cellular distribution. Comparable field in a typical AD case shows cytoplasmic accumulation and nuclear loss of DNMT1 immunoreactivity (a2). Figures (c1) and (c2) show normal nuclear immunoreactivity for both ND and AD respectively in the pathologically spared cerebellum. Similar patterns of immunoreactivity were observed for RNA pol2 in ND (b1) and AD (b2) in CA1 of hippocampus and ND (d1) and AD (d2) in cerebellum. (Scale bars = 15 µM). (e) Quantification of the cellular distribution of immunoreactivity in CA1 of the hippocampus in 5 AD and 5 ND samples. 100 neurons from CA1 of AD and ND cases were quantified and the mean signal/group/area (i.e. signal intensity/AD or ND/nuclear, cytoplasmic or both) was analyzed. Neurons were evaluated by delineating the nucleus (dashed circle) from cytoplasm (solid black line). (f) DNMT1 and RNA pol II affymetrix array data from AD (n = 10) and ND (n = 10) hippocampal CA1 neurons (500 neurons/case). * Indicates significant difference compared to control, 2-tailed t-test p = <.05.

Fluorescence and bright field intensity analysis was performed using the Image J software. The intensity measurements were corrected for background differences by dividing the measured intensities with the average intensity of a region that showed no reactivity. Per image, the fluorescence intensity of 20 individual neurons per sample per condition, totaling 100 neurons from AD and 100 neurons from ND were analyzed. Neurons were evaluated by delineating the nucleus (dashed circle) from cytoplasm (solid black line, see figure 1), of each cell and measuring the mean intensity value of each area (i.e. nuclear and cytoplasmic). Analysis of fluorescence intensities were calculated in arbitrary units and do not represent the absolute quantity of these markers. Significance was determined using a two-tailed, paired student’s t-test and declared significant at a p-value <.05. Correlation analysis was determined by using Pearson product-moment correlation coefficient analysis. Significance was set at the.05 level.

#### Array

A two-tailed unpaired t-test, assuming unequal variance (using multiple testing corrections by Benjamini and Hochberg False Discovery Rate), was applied to identify genes whose expression was significantly different in AD and ND. Fold changes were calculated by taking the ratio of the average expression signal for a particular gene across all samples (ND and AD) divided by the average expression signal for the same gene in ND samples (see [14]for further details).

## Results

### Reduced Nuclear Localization and Cytoplasmic Sequestration of DNMT1 and RNA Pol II in CA1 Neurons of Human AD Hippocampus

Both epigenetic and transcription-related nuclear proteins evaluated, DNMT1, and RNA pol II exhibited markedly reduced nuclear immunoreactivity in AD hippocampal CA1 neurons compared to matched, cognitively normal ND cases. In AD, only very faint, punctate, nuclear immunoreactivity could be observed, whereas cytoplasmic staining was readily apparent. The cytoplasmic accumulation of nuclear proteins was largely contained within the cell soma, with some extension to first order dendrites and less extension to distal dendrites (Figure 1, a2, b2). ND neurons, by contrast, showed profuse immunoreactivity for both DNMT1 and RNA pol II within the nuclear compartment, with only modest cytoplasmic accumulation (Figure 1, a1, b1 respectively). Pathologically spared cerebellum was virtually identical in nuclear immunoreactivity in both ND and AD samples (Figure 1, c1–c2, d1–d2). Quantitation of AD neurons within CA1 (Figure 1e) showed significant decrease in DNMT1 nuclear immunoreactivity p = 8.5e-7 (t-test), and for RNA pol II p = 1.2e–4 (t-test). Cytoplasmic immunoreactivity for DNMT1 was significantly increased in AD, p = 1.8e–10 (t-test), and for RNA pol II, p = .001 (t-test). Moreover, correlation analysis between nuclear and cytoplasmic immunoreactivity revealed a significant negative correlation (r = −.45, p = .006 for DNMT1, and r = −.27, p = .012 for RNApol II) in AD neurons. Thus, as nuclear immunoreactivity decreases, cytoplasmic immunoreactivity increases. Similar regression analysis of control samples showed no significant correlation for DNMT1 (r = .12, p = .234) or RNA pol II (r = .22, p = .19). Array analysis of transcript expression of ten Alzheimer’s cases and ten well matched controls showed decreased expression of both DNMT1 and RNA pol II (Figure 1f). Although both mRNA probes show decreased expression in the CA1 of hippocampus, RNA pol II was the only probe to reach significance p = .0029 (t-test).

### Decreased Neuronal RAN mRNA and Protein in AD CA1 of Human Hippocampus

**Figure 2:**
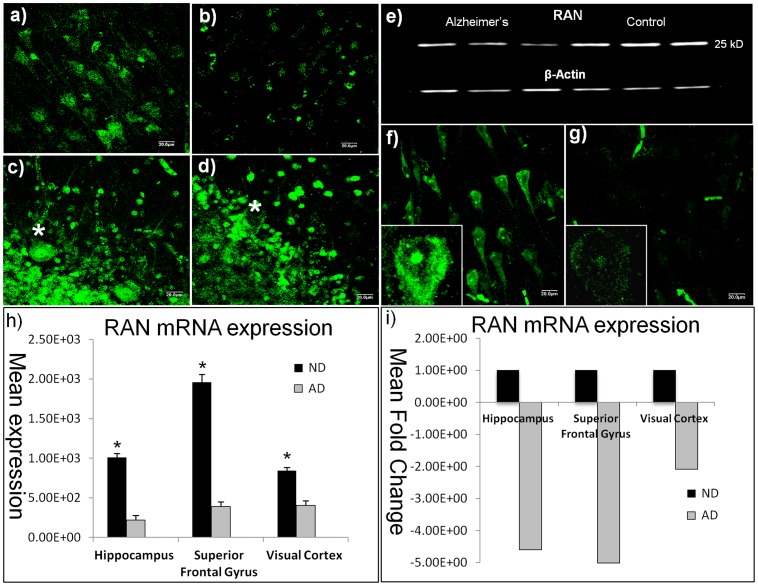
Decreased Ran protein and message in CA1 of AD hippocampus. Confocal micrographs (488 nm bandwidth, green fluorphore) reveals robust nuclear and cytoplasmic RAN immunoreactivity in ND hippocampal CA1 neurons (a), compared to AD (b). Pathologically spared cerebellum show similar patterns of immunoreactivity in both ND (c) and AD (d) samples in all but the larger Purkinje neurons (asterisks). (e) Significant AD decrements in RAN protein were confirmed in Western blot analyses (p<0.0001). (f) 40X micrographs show normal cytoplasmic and nuclear distributions of RAN message by fluorescence *in situ* hybridization in ND. By contrast, there was a significant decrease (p = 0.00001) in overall signal in comparable fields from AD, with limited reactivity in the cytoplasm and slightly greater reactivity in the nucleus (g). (h) Mean RAN expression, and fold change (i) from Affymetrix array data looking at AD (n = 10) and ND (n = 10) hippocampal CA1 neurons (500neurons/case). *Indicates significant difference between AD and control at p<0.05. Scale bars = 15 µm.

To investigate potential mechanisms that might underlie reduced nuclear and increased cytoplasmic localization in AD neurons, we assessed the expression of the key nucleocytoplasmic transport molecule, RAN. In ND hippocampus, RAN immunoreactivity was abundant, and localized throughout the nuclear compartment and cell soma, extending into the apical dendrites (Figure 2a). This is the appropriate distribution for RAN [15], a molecule whose function requires it to be transiently present in the cytoplasm or nucleus depending on the direction of transport. In AD cases, however, RAN immunoreactivity was qualitatively decreased, with the limited amount of staining being primarily localized to the nucleus (Figure 2b). Western blots of AD and ND cortical grey matter from temporal neo cortex quantitatively confirmed reduced protein expression, showing significant RAN decreases (p = 0.0001) in AD compared to ND samples (Figure 2e). RAN deficits in AD were negligible in the pathologically-spared cerebellum, although an occasionally void in immunoreactivity could be seen in the larger Purkinje neurons in AD (see asterisk, Figure 2c, 2d).

At the transcript level, analysis of Affymetrix Human Genome U133 Plus 2.0 microarray data showed significant differences in RAN mRNA expression in AD compared to ND in laser captured neurons from CA1 of hippocampus, superior frontal gyrus, and primary visual cortex (Figure 2 h and i). Fold changes were similar in the heavily-impacted hippocampus and superior frontal gyrus (−4.6 and −5.0, respectively, for AD relative to ND case), and were substantially less (−0.25) in the primary visual cortex, a cortical region with only modest AD pathology [16] (Figure 2 j and k). In addition to RAN mRNA, transcript expression for several key RAN binding proteins was analyzed and found to exhibit significant AD deficits (Table 2).

**Table 2:** mRNA expression from laser captured neurons in the CA1 of the hippocampus, superior frontal gyrus, and visual cortex using Affymetrix Human Genome U133 plus 2.0 microarrays.

Brain Region	Gene Name	Description	p-value	Fold Change	Mean ND	Mean AD
	RAN	RAN, member RAS oncogene family				
Hippocampus			5.82E−05	−4.60E+00	1.01E+03	2.20E+02
Superior Frontal Gyrus			1.32E−02	−5.01E+00	1.96E+03	3.91E+02
Visual Cortex			1.97E−02	−2.08E+00	8.43E+02	4.05E+02
	RANBP1	RAN binding protein 1				
Hippocampus			2.35E−03	−2.47E+00	3.02E+02	1.22E+02
Superior Frontal Gyrus			2.74E−02	−2.57E+00	3.83E+02	1.49E+02
Visual Cortex			3.85E−01	−1.28E+00	1.90E+02	1.48E+02
	RANBP2	RAN binding protein 2				
Hippocampus			7.14E−03	−1.74E+00	7.96E+02	4.57E+02
Superior Frontal Gyrus			6.12E−02	−1.91E+00	5.83E+02	3.06E+02
Visual Cortex			4.15E−02	−1.22E+00	3.73E+02	3.06E+02
	RANBP5	RAN binding protein 5				
Hippocampus			4.21E-02	−1.49E+00	1.69E+02	1.14E+02
Superior Frontal Gyrus			2.26E−02	−2.86E+00	2.65E+02	9.26E+01
Visual Cortex			8.82E−04	−1.79E+00	1.62E+02	9.07E+01
	RANBP6	RAN binding protein 6				
Hippocampus			8.21E+00	1.03E+00	1.15E+03	1.19E+03
Superior Frontal Gyrus			2.05E−02	−3.02E+00	3.05E+03	1.01E+03
Visual Cortex			3.94E−02	−1.47E+00	1.38E+03	9.39E+02
	RANBP9	RAN binding protein 9				
Hippocampus			6.26E−05	−3.06E+00	1.89E+02	6.18E+01
Superior Frontal Gyrus			1.57E−02	−2.62E+00	1.98E+02	7.56E+01
Visual Cortex			1.71E−01	−1.45E+00	1.05E+02	7.29E+01
	RANBP10	RAN binding protein 10				
Hippocampus			1.74E−02	−2.07E+00	1.42E+02	6.86E+01
Superior Frontal Gyrus			1.64E+00	−1.36E+00	1.11E+02	8.14E+01
Visual Cortex			1.14E+00	−1.19E+00	8.79E+01	7.37E+01

mRNA data from all three brain regions show a significant decreases in RAN and RAN binding proteins, with lesser amounts in the visual cortex, an area with only modest AD pathology [16]. With the exception of RANBP6 in the hippocampus, all other RAN binding proteins were significantly down in AD compared to controls.

Reduced RAN transcript in AD was also demonstrated by fluorescence-label *in situ* hybridization. Signal for RAN mRNA was strongly localized to the nucleus, nucleolus, and cytoplasm of ND hippocampal CA1 neurons (Figure 2f), but was negligible in AD neurons (Figure 2g). Although the reactivity was weak in AD, the most prominent labeling could be observed within the nuclear compartment, with limited cytoplasmic signal that was consistent with the findings from immunohistochemistry (c.f., Figure 2b). Both AD and ND samples were incubated with sense probes and showed no obvious immunoreactivity (data not shown).

### Reduced Nuclear Localization of Epigenetic and Other Transcription-related Molecules in Oligomeric Aβ42 Treated SK-N-BE(2) Neuroblastoma Cells

**Figure 3:**
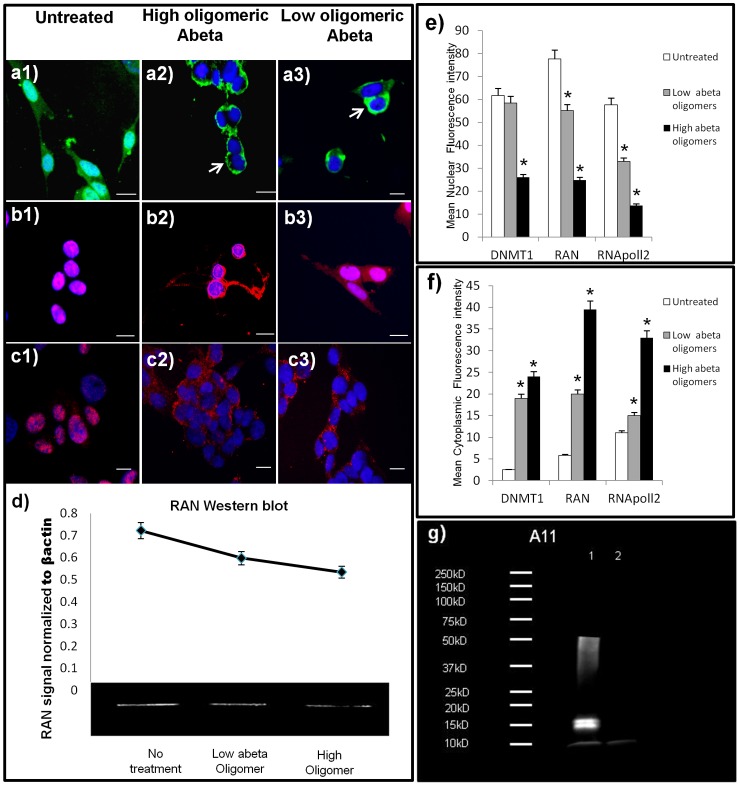
*In vitro* Aβ42 treatment replicates human neuronal distributions of nuclear proteins. Micrographs of DNMT1 and RNA pol II in neurons (SK-N-Be(2)) treated with higher molecular weight (MW) oligomers of Aβ42 (1 uM) or lower MW oligomers of Aβ42 (1 uM) for 36 hours. High power micrograph (40X) of cultures labeled with an antibody to RAN before (a1) and after treatment with higher MW oligomeric Aβ42 (a2) or lower MW oligomeric Aβ42 (a3); shows nuclear and cytoplasmic loss with nuclear envelope accumulation (arrows), similar to that seen *in vitro*. Western blot analysis confirms these data of an overall reduction in basal RAN protein levels when treated with oligomeric abeta (d). Normal distributions for nuclear molecules DNMT1 (b1), and RNA pol II (c1) was readily apparent in the nucleus of untreated neurons, but translocation to the cytoplasm is seen in both molecules when treated with high or lower MW oligomeric Aβ42; Dnmt1 (b2, b3), and RNA pol II (c2, c3). e) Mean nuclear fluorescence intensity and mean cytoplasmic fluorescence intensity (f) of nerve cells treated with either low MW Aβ42 oligomers, or high MW Aβ42 oligomers. Asterisk (*) signifies a significant difference compared to control samples (p<0.05). Data are presented as mean +/− S.E.M. (g) Western blot analysis using oligomeric antibody A11, revealed the presence of oligomers in both preparations, with higher MW oligomers in lane 1 (96 hour aggregation), compared to lane 2 (immediately frozen). (Scale bars = 15 um).

Since disrupted nuclear localization was observed in AD in which amyloid precursor protein is expressed in excess, we confirmed the relationship between Abeta and RAN expression experimentally by treating cells with Abeta *in vitro*. Experimental treatment of neuroblastoma cells with 1 µm oligomeric Aβ42 corroborated our hypothesis that amyloid is involved in reduced expression of RAN and consequent aberrant cytoplasmic sequestering of DNMT1 and RNA pol II. Figure 3, a1 shows the normal distribution of RAN protein in untreated cells and the aberrant loss of nuclear and cytoplasmic RAN with higher MW oligomeric Aβ42 (Figure 3, a2) and lower MW oligomeric Aβ42 treatment (Figure 3, a3). Although both species of Aβ42 were found to reduce RAN protein, higher MW oligomers was found to reduce RAN 19% more than lower MW oligomers (Figure 3d). Both epigenetic and transcription-related nuclear proteins evaluated, DNMT1, and RNA pol II, showed qualitative and quantitative decreases in nuclear immunoreactivity and increased cytoplasmic sequestering when treated with both lower and higher MW oligomeric Aβ42 (Figure 3e and 3f). All experiments were performed in triplicate.

### Knock Down of RAN Message by SMARTpool siRNA in vitro Induces Cytoplasmic Sequestering of Nuclear Proteins Similar to that Observed in situ in the AD Brain

**Figure 4:**
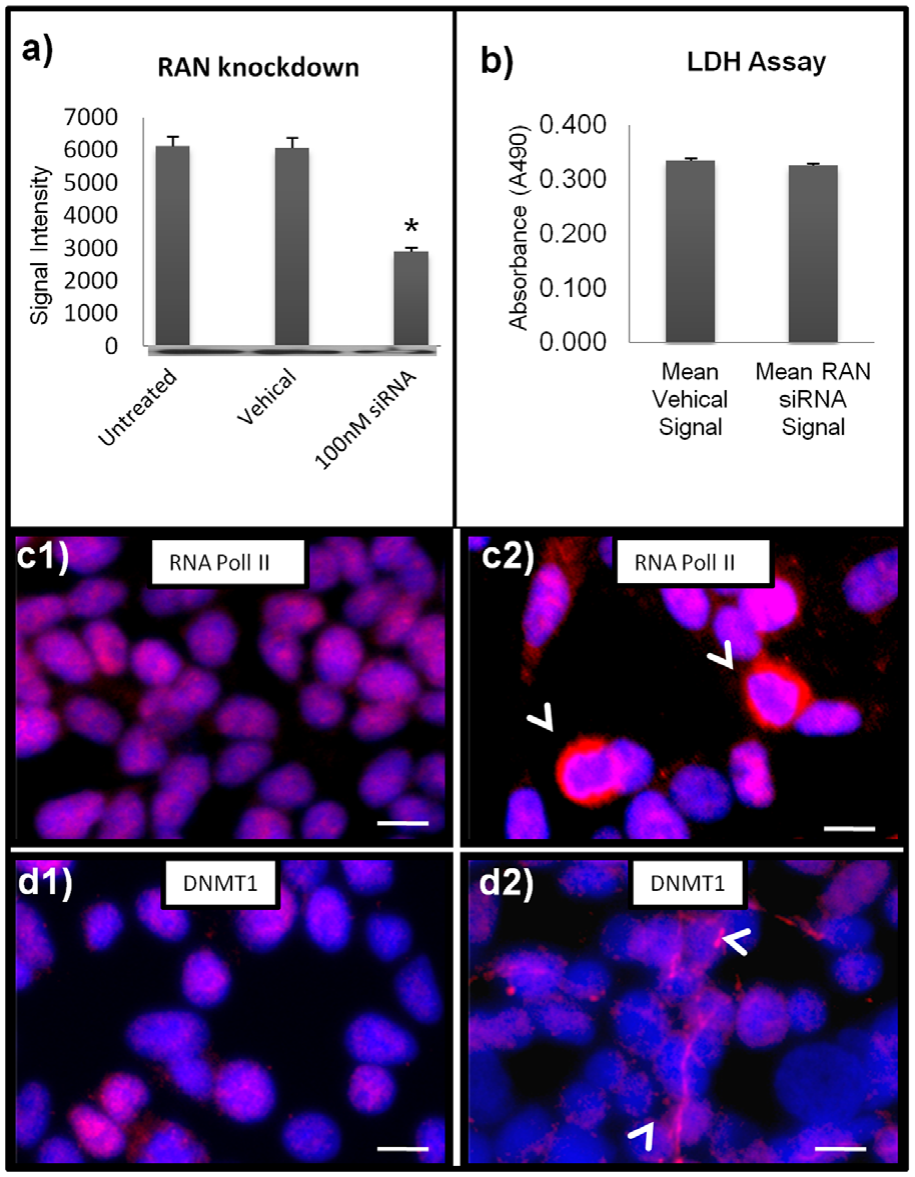
RAN knock down *in vitro* replicates neuronal distribution of nuclear proteins. Neuroblastoma cells were treated with 100 nM RAN SNArpol siRNA, a concentration that reduced RAN levels by 71% (a), but did not materially affect cell viability (b). Untreated, tranfection reagent only (c1, d1) and treated, RAN siRNA (c2–d2) cells were immunoreacted for RNA polII (c1, c2), and DNMT1 (d1, d2). After 48 hours, RAN knockdown induced cytoplasmic accumulation and nuclear losses in both target proteins, recapitulating *in vivo* observations in AD brain. DNMT1 IR after siRNA treatment was largely localized in axons (d2, arrows), RNA pol2 immunoreactivity was chiefly located in the cytosol (c1, arrows).

To further validate a link between aberrant localization of nuclear proteins in AD neurons and RAN deficits, we conducted RAN knockdown experiments in SK-N-BE(2) neuroblastoma cells using an experimentally-determined optimum concentration of RAN siRNA of 100 nM. After knock down, Western blots of RAN protein showed a mean RAN reduction of 71% (Figure 4a). Prior to exposure, SK-N-BE(2) exhibited normal nuclear localization of RNA pol II and DNMT1 (Figure 4, c1 and d1 respectively), but this pattern was disrupted after 48 hours of RAN siRNA treatment (Figure 4, c2 and d2). Nuclear localization of RNA pol II, for example, was reduced, whereas abnormal accumulation in the cytoplasm was readily apparent, similar to the pattern observed in AD neurons (compare Figure 4 c2, with Figure 1, b2).

## Discussion

Our data demonstrate in Alzheimer’s disease greatly diminished nuclear localization and increased cytoplasmic localization of two molecules representative of two different classes of mechanisms in the regulation of expression: DNMT1, an epigenetic molecule modulating DNA methylation, and RNA pol II, central to transcription. The diminished nuclear localization is demonstrated in brain regions conventionally affected in AD but not in regions traditionally less affected in AD. We show this altered nuclear/cytoplasmic ratio associated with reduced expression of RAN. We also demonstrate reduced expression of RAN and altered nuclear/cytoplasmic localization in a mouse model of AD. Since both human AD and the mouse model of AD are associated with expression of Abeta, we demonstrate that *in *vitro treatment with Abeta results in both reduced expression of RAN and aberrant nuclear and cytoplasmic localization of DNMT1 and RNA pol II. In order to separate the effect of reduced RAN expression from effects of Abeta we used siRNA to knock down of RAN expression *in vitro* and showed that this resulted in reduced nuclear and increased cytoplasmic localization of DNMT1 and RNA pol II.

### Miss-localization of Transcriptional Regulators

Failure of nuclear proteins to reach the sites at which they act would have many pathogenic consequences. For example, nuclear loss and cytoplasmic sequestering of DNMT1 has been linked to increased α-synuclein in Parkinson’s disease, Lewy body disease, and α-synuclein transgenic mice [17], and has been suggested to underlie global DNA hypomethylation in affected neurons similar to that which we previously reported in AD [12], [18]. More globally, inadequate transport of epigenetic and other nuclear proteins would be expected to disrupt chromatin structure, which could lead to large-scale changes in gene expression such as have been described in microarray studies of AD brain where thousands of genes mediating dozens of essential cell functions are altered [14], [19]–[21].

In addition to the pathogenic potential of reduced presence of transcriptional regulators in the nucleus, their aberrant presence in the cytoplasm may also have deleterious consequences on cytoskeletal elements, mitochondria, and other organelles. For example, Husseman and colleagues observed that cytoplasmic phosphorylated RNA polII co-localized with cdc2, a cell cycle enzyme that phosphorylates and inhibits RNA pol2, an event that was suggested to precede neurofibrillary tangle formation [22]. Other reports have shown that other epigenetic factors such as extracellular HDAC1 disrupts axonal transport by interacting with motor proteins, leading to axonal loss [23].

### The Involvement of Amyloid

The role of Aβ42 in disrupted nuclear transport suggested by the abundance of amyloid pathology in the vulnerable CA1 of the hippocampus, is further supported by our data showing that Aβ42 treatment of neurons mimics aspects of our human data in spite of the time course differences in Aβ42 exposure. We certainly do not suggest that APP is the only potential modulator of nuclear transport, as it is known, for example, that reactive oxygen species may also modulate nuclear transport [9]. The relative contributions of APP and/or its fragments, reactive oxygen species as well as other modulators of transport on the AD cellular phenotype remain to be tested in detail. Our study however, demonstrates a relationship among Aβ42 treatment, loss of RAN, and cytoplasmic sequestering of nuclear proteins. Although it remains to be determined what portion of APP may be responsible for the effects reported *in vivo*, our data implicate oligomeric Aβ42 in knocking down RAN protein and subsequently reducing nuclear localization.

An additional aspect of reduced expression of RAN may be its effect on the synapse through activation of caspase 3. *In vitro* analysis of RAN knockdown has shown been shown to activate caspase 3 [24], both a potent activator of apoptosis [25] and also a trigger of early synaptic dysfunction [26]. Activated caspase 3 has been implicated in multiple effects seen in AD, including GGA3 cleavage, which is required for BACE lysosomal degradation [27], the PI3k-Akt/mTOR pathway which regulates Aβ oligomer induced neuronal cell cycle events [28] and tau hyperphosphorylation, which cleaves tau and initiates/or accelerates tau pathology [29]. Microarray data indicate a strong positive correlation of gene expression between RAN and synaptophysin, r = 0.85 (, p = 0.000005) for AD and r = 0.82 p = 0.00001) for age matched controls. Collectively, these data suggest a cascade of events which includes the down-regulation of RAN by Aβ42 oligomers (shown here), the release of caspase 3 in response [24], and resulting degeneration of synapses [26].

Whether the many other nuclear proteins that rely on RAN-mediated nucleocytoplasmic transport follow similar pattern we observed in DNMT1 and RNA pol II remains to be determined. If they do, however, deficits in RAN followed by ectopic intracellular localization of epigenetic and other transcription-related molecules could well be a central event in the pathophysiology of AD, and could provide an overarching, integrative mechanism for the myriad pathogenic processes that occur in the disorder. Moreover, it is important to note, that the current study focused solely on neuronal populations and non-neuronal cells (i.e. glia) could prove to be similarly affected.

In closing, our data suggest a model in which ectopic intracellular localization of epigenetic molecules may be a central event in the pathophysiology of AD. This model, driven by Aβ42 resulting in RAN depletion, has several components that suggest broader implications. These components are: 1) Failure of epigenetic molecules to translocate to the nucleus in affected neurons in AD, with consequential effects on chromatin structure and, as a result, gene expression. 2) Increased epigenetic molecules in the cytoplasm which may lead to detrimental interactions with cytoskeletal elements, an insult in transport along neuronal processes, mitochondria and other organelle dysfunction, affecting structural and functional cellular properties.

## Supporting Information

Figure S1 The general health of the neurons in culture was tested by performing two tests to ensure the cultures were viable and we’re not undergoing apoptosis. 1) LDH assay, and 2) Calcein AM treatment.
(TIF)
